# Circadian Rhythms in Floral Scent Emission

**DOI:** 10.3389/fpls.2016.00462

**Published:** 2016-04-13

**Authors:** Myles P. Fenske, Takato Imaizumi

**Affiliations:** Department of Biology, University of Washington, SeattleWA, USA

**Keywords:** floral volatile, circadian clock, pollinator, metabolic pathway, petunia, tobacco, *Solanaceae*

## Abstract

To successfully recruit pollinators, plants often release attractive floral scents at specific times of day to coincide with pollinator foraging. This timing of scent emission is thought to be evolutionarily beneficial to maximize resource efficiency while attracting only useful pollinators. Temporal regulation of scent emission is tied to the activity of the specific metabolic pathways responsible for scent production. Although floral volatile profiling in various plants indicated a contribution by the circadian clock, the mechanisms by which the circadian clock regulates timing of floral scent emission remained elusive. Recent studies using two species in the *Solanaceae* family provided initial insight into molecular clock regulation of scent emission timing. In *Petunia hybrida*, the floral volatile benzenoid/phenylpropanoid (FVBP) pathway is the major metabolic pathway that produces floral volatiles. Three MYB-type transcription factors, ODORANT 1 (ODO1), EMISSION OF BENZENOIDS I (EOBI), and EOBII, all of which show diurnal rhythms in mRNA expression, act as positive regulators for several enzyme genes in the FVBP pathway. Recently, in *P. hybrida* and *Nicotiana attenuata*, homologs of the *Arabidopsis* clock gene *LATE ELONGATED HYPOCOTYL* (*LHY*) have been shown to have a similar role in the circadian clock in these plants, and to also determine the timing of scent emission. In addition, in *P. hybrida*, PhLHY directly represses *ODO1* and several enzyme genes in the FVBP pathway during the morning as an important negative regulator of scent emission. These findings facilitate our understanding of the relationship between a molecular timekeeper and the timing of scent emission, which may influence reproductive success.

## Introduction

Pollinator attraction is a critical event for many flowering plants that require out-crossing. Flowers and related structures (e.g., bracts) are primarily responsible for attracting pollinators, through variations in shape, color, and scent. In addition, the traits that determine the volume of nectar (which rewards pollinator visits) and the position of reproductive organs (which influence the transfer of pollen to other plants) also affect the success of pollination (Sheehan et al., 2012). These floral traits are temporally regulated in many species. As captured by Linnaeus’ widely known flower clock, many flowers open and close at particular times of the day (Rose, 1775).

Among the traits related to pollinator syndrome, floral scent is a critical component of ensuring successful pollination for many plant species (Dudareva et al., 2006). Over 90% of global angiosperms, and about a third of crop species, require pollination by animals (mostly insects; McGregor, 1976; Kearns et al., 1998; Klein et al., 2007). Pollinating insects have well developed sensory mechanisms for detecting floral odors, and like most organisms, insects exhibit daily fluctuations in their activities (Saunders, 1997). Plants maintain their side of this temporal relationship by emitting scent only during specific times of day, which corresponds with the activity periods of the pollinators. This timing of scent release likely allows for efficient resource utilization, as well as limiting the visibility of the plant to herbivores. In this review, we discuss the brief history of the findings that elucidate the influence of the circadian clock on scent emission as well as recent findings of the molecular links in between.

## Observations of Rhythmic Scent Release

An early scientific documentation of scent rhythmicity comes from Lillian Overland’s research at the Missouri Botanical Garden, where she measured the presence of floral fragrance throughout the day (Overland, 1960). She used *Cestrum nocturnum*, commonly known as “night blooming jasmine,” a member of the *Solanaceae* family. *C. nocturnum*’s small tube-shaped flowers open and emit a strong, pleasant scent at night. At the time, it was assumed that the emission from *C. nocturnum* was the result of a nighttime-dependent mechanism, rather than circadian control of the rhythm. Until the mid-1950s, the presence of an endogenous oscillator (i.e., the circadian clock) and the importance of its influence on biological responses was not widely accepted. Since then, a significant percentage of physiology has been attributed to circadian clock regulation.

Overland hypothesized that *C. nocturnum’s* nocturnal scent emissions were under the regulation of this endogenous timekeeper. Flowering plants were placed into rooms with continuous light and temperature, and the scent presence was recorded over 3 days. Under the conditions, a clear 24-h rhythm was observed, with scent occurring during the time periods corresponding with what would be night (subjective night). This work clearly demonstrated that *C. nocturnum* plants utilized the circadian clock to time floral scent emission. In addition, the cell autonomous nature of the clock was captured by demonstrating that detached corolla lobes emitted scent in a circadian fashion in the same way as that of the intact flower.

## Technical Improvement in Floral Scent Measurement

Gas chromatography–mass spectrometry (GC–MS) would prove to be a powerful and more quantitative tool for scientists who study flower fragrance, perhaps first used to measure scent periodicity in orchids in 1978 (Nilsson, 1978). Altenburger and Matile (1988, 1990) and Matile and Altenburger (1988) released a series of three papers between 1988 and 1990 that provided thorough analyses of scent rhythmicity using modern chemical analysis and time course scent collection techniques. An automated collection device was created to facilitate the consecutive harvest of scent samples during extended time courses, and GC–MS was used to analyze the sequential changes of scent release.

In their first paper, diurnal rhythms of different volatiles were observed in four species: *Odontoglossum constrictum, Citrus medica, Hoya carnosa*, and *Stephanotis floribunda* (Matile and Altenburger, 1988). *O. constrictum* and *C. medica* exhibited emission peaks during the day, while *H. carnosa* emitted scent only at night. The timing of the five most abundant compounds emitted from the flowers of *H. carnosa* were notably synchronized, indicating a similar mechanism of regulatory timing. The emission profile of *S. floribunda* was even more intriguing, as its flower emitted separate compounds at different times of the day. The emission of methyl benzoate and linalool peaked at midnight, but the third compound, 1-nitro-2-phenylethane, peaked at noon – directly antiphasic to the first two. This phenomenon raised several interesting points. First, separate mechanisms must set the phase of each emission. In addition, while the primary purpose of methyl benzoate and linalool is attracting nocturnal pollinators, the objective of 1-nitro-2-phenylethane release is unknown. Is 1-nitro-2-phenylethane involved in attracting a separate pollinator during the day, or does it fill an entirely different purpose, perhaps as a repellent? It should be mentioned that methyl benzoate and 1-nitro-2-phenylethane are both components of the benzenoid metabolic pathway, while linalool is a member of the terpenoid pathway (Silva et al., 2006; Muhlemann et al., 2014).

Their second paper provided evidence of circadian clock involvement, as floral scent rhythms were maintained even under continuous light conditions in *H. carnosa* (Altenburger and Matile, 1988). After shifting the photoperiod by 12 h, the plants were able to quickly entrain to the new light/dark cycles. Another critical finding was that flowers separately entrained to 12-h shifted (antiphasic) light/dark cycles from the vegetative body (which they were still attached to) properly entrained to their own surrounding photoperiods, demonstrating that flowers are able to generate their own circadian rhythms independent to the rest of the plant. Similar to the previously described example of detached *C. nocturnum* corolla lobes, this implied the presence of tissue/cell autonomous circadian timekeeper mechanisms in flowers. Cut flowers of *H. carnosa* exhibited nearly undetectable levels of scent emission (Matile and Altenburger, 1988), indicating that, in some plants, inter-tissue connection (i.e., between leaves and flowers) is necessary for floral scent emission.

The third paper elaborated upon the rhythmic scent emissions of the species introduced in the first paper (Altenburger and Matile, 1990). *H. carnosa*’s emissions exhibited temperature compensation in the free-running period, and were able to maintain a circadian rhythm in continuous dark. The emission of *S. floribunda* volatiles in continuous light was found to be circadian in nature, yet they quickly fade into arrhythmicity in just 2 or 3 days. Cut flowers of *O. constrictum* and *C. medica* were unable to maintain a rhythmic emission under both continuous light and dark conditions, but were able to resume a rhythm when returned to diurnal light/dark cycles.

A separate group completed similar experiments using GC–MS analysis. Loughrin et al. (1991) analyzed the daily emission of volatiles in tobacco flowers (*Nicotiana sylvestris* and *N. suaveolens*). They analyzed the volatile emission patterns under continuous light conditions, and found that the emission of benzyl alcohol and methyl benzoate, the most abundant volatiles in their flowers, occurred in a clear circadian fashion. Interestingly, under the same conditions, emission of the sesquiterpene hydrocarbon, caryophyllene, in *N. sylvestris* did not show any oscillation. This result showed that, even in the same flower, the influences of circadian timing are different depending on the metabolic pathways/products.

These four examples tell us that, even though all of these plants show robust daily scent emission rhythms, the contribution of their circadian clocks to scent emission under continuous environmental conditions varies depending on the species.

The scent model *P. hybrida* exhibits a distinct scent emission at night, but its robust oscillation does not persist in continuous light or dark (Underwood et al., 2005; Fenske et al., 2015). Interestingly, *P. axillaris* (one parent of *P. hybrida*) maintains a robust oscillation of scent release in continuous light, whereas *P. integrifolia* (the other parent) does not (Hoballah et al., 2007). Hybridization between *P. axillaris* and *P. integrifolia*’s light-dependent scent patterns may attribute to *P. hybrida*’s non-robust scent oscillation under continuous light conditions.

## Models of Scent Emission

By the 21st century, several model plant species had arisen in the scent biology field, the most prolific of which were rose, snapdragon, tobacco, and petunia. Choosing models to study scent was important to develop a comprehensive understanding of scent production. A focused study of the metabolic pathways that produce scent was a necessary prerequisite to understanding the regulation of scent emission. In this regard, *P. hybrida* has emerged as perhaps the most complete model in scent emission, as the majority of the metabolic pathway has been mapped, and transcriptional regulators of floral scent have been characterized (Dudareva and Pichersky, 2006; Colquhoun and Clark, 2011).

## Floral Volatile Genes Have Daily Oscillatory Expression Patterns

An analysis of gene expression within the floral volatile benzenoid/phenylpropanoid (FVBP) pathway provided key insights into the mechanism of scent regulation (Dudareva et al., 2000). Emission of the major volatile methyl benzoate diurnally oscillates over the course of several days in snapdragon, in both continuous light and darkness (Kolosova et al., 2001). The daily changes in supply of the substrate of methyl benzoate (benzoic acid) largely accounts for the circadian oscillation (Kolosova et al., 2001). Kolosova et al. (2001) analyzed the expression of benzoic acid carboxyl methyltransferase (*BAMT*) and phenylalanine ammonia-lyase (*PAL*) genes. BAMT catalyzes the final step for methyl benzoate synthesis by transferring a methyl group to benzoic acid (Dudareva et al., 2000). PAL controls benzoic acid synthesis by producing *trans*-cinnamic acid from the amino acid phenylalanine (Colquhoun and Clark, 2011). The snapdragon *BAMT* and its petunia homologs *BSMT1* and *BSMT2*, all exhibit diurnal oscillations, peaking in the afternoon. However, the *BSMT1* and *BSMT2* expression patterns do not directly correlate with the volatile expression patterns (Kolosova et al., 2001; Fenske et al., 2015). *PAL* mRNA expression (and enzymatic activities) also oscillates in diurnal conditions in both snapdragon and petunia; however, interestingly, it peaks at different times (around dawn in snapdragon, dusk in petunia). *PAL* mRNA expression oscillates in a similar pattern to that of benzoic acid synthesis in diurnal conditions (Kolosova et al., 2001; Fenske et al., 2015). These results support the notion that diurnal emission patterns of scent largely derive from the oscillation of upstream metabolite synthesis, rather than controlling the final reaction timing.

**FIGURE 1:**
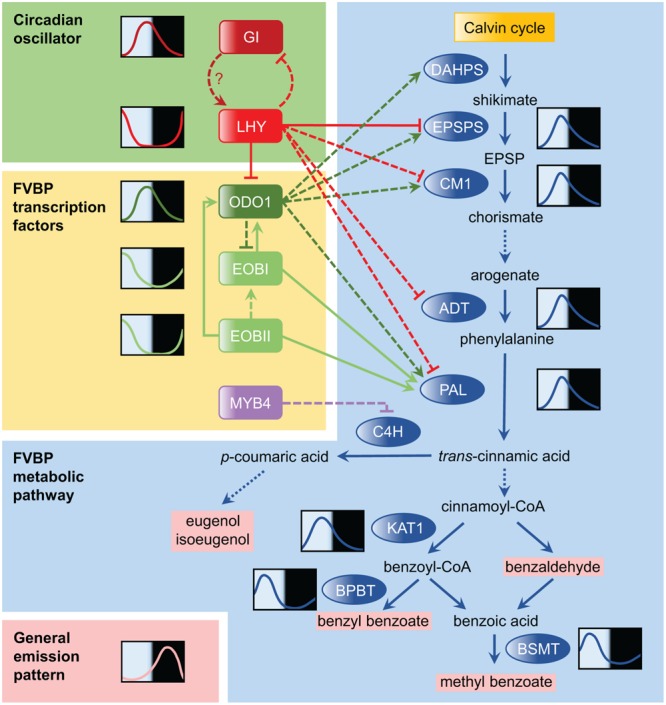
**Temporal regulation of the floral volatile benzenoid/phenylpropanoid (FVBP) pathway in petunia.** The FVBP pathway is the primary metabolic pathway of petunia for scent production. The FVBP pathway is composed of a series of enzymes (shown in blue ovals), which includes 3-deoxy-D-arabino-heptulosonate-7-phosphate synthase (DAHPS), 5-enolpyruvylshikimate-3-phosphate synthase (EPSPS), chorismate mutase 1 (CM1), arogenate dehydratase (ADT), phenylalanine ammonia-lyase (PAL), 3-ketoacyl-CoA thiolase (KAT1), benzoyl-CoA:benzyl alcohol/phenylethanol benzoyltransferase (BPBT), *S*-adenosyl-L-methionine:benzoic acid/salicylic acid carboxyl methyltransferase (BSMT), and cinnamate 4-hydroxylase (C4H). These enzymes modify products of the Calvin cycle into volatile scents that are emitted by the flowers (volatile products shown in pink). Solid lines in the FVBP pathway represent single enzymatic steps, while dotted lines represent multiple steps. Representative daily mRNA expression patterns are drawn for selected genes. The relationship between LATE ELONGATED HYPOCOTYL (LHY) and GIGANTEA (GI) in the circadian oscillator was conceptualized based on knowledge obtained from *Arabidopsis* research and their expression patterns in petunia. Transcriptional regulators in the FVBP pathway are highlighted by yellow backgrounds, including ODORANT 1 (ODO1), EMISSION OF BENZENOIDS I (EOBI), EOBII, and MYB4. Solid arrows connecting transcription factors to FVBP genes denote confirmation of direct molecular binding demonstrated by electrophoretic mobility shift assay (EMSA), yeast one-hybrid, and/or transient reporter assay; dashed lines lack this confirmation. EOBI may regulate the FVBP gene expression more directly (not through the regulation of *ODO1*). The general emission pattern of benzenoid compounds is shown in the bottom left-hand corner; this profile is synthesized based on daily scent emission profiles of four major benzenoids in Fenske et al. (2015).

Among the other FVBP enzyme genes in petunia, genes encoding 3-deoxy-D-arabino-heptulosonate-7-phosphate synthase (DAHPS), 5-enolpyruvylshikimate-3-phosphate synthase (EPSPS), arogenate dehydratase (ADT), chorismate mutase 1 (CM1), eugenol synthase (EGS), isoeugenol synthase (IGS), 3-ketoacyl-CoA thiolase (KAT1), benzoyl-CoA:benzyl alcohol/phenylethanol benzoyltransferase (BPBT), and *S*-adenosylmethionine synthetase (SAMS) also exhibit robust diurnal oscillations in their expression (Verdonk et al., 2005; Fenske et al., 2015) (**Figure 1**), though most show only weak oscillations in continuous dark (none do in continuous light). At least in *P. hybrida*, it appears likely that robust scent emission cycles are derived from the cumulative effect of the oscillatory expression of many FVBP genes in concert.

## Regulatory Mechanisms of the Scent Metabolism Pathway

The first regulatory component of floral scent metabolism was identified as ODORANT1 (ODO1), an R2R3-type MYB transcription factor in *P. hybrida* (Verdonk et al., 2005). mRNA expression of *ODO1* peaks in the evening, just prior to scent release. RNAi suppression of *ODO1* transcript caused a significant reduction in emission of nearly all detectable volatile benzenoids. In addition, *ODO1* suppression drastically reduced the transcript levels of many key FVBP genes, including *DAHPS, EPSPS, PAL, CM*, and *SAMS* (Verdonk et al., 2005). As the authors note, many of these enzymes are upstream in the FVBP metabolic pathway, which poses the question: does *ODO1* affect more than just scent emission? *ODO1* suppression did not affect accumulation of anthocyanin, which is produced through the FVBP pathway in flower petals (Verdonk et al., 2005). This indicated that while ODO1 controls this pathway broadly, its influence may be more specific to volatile production.

EMISSION OF BENZENOIDS II (EOBII) was the second regulatory component found to be involved in FVBP synthesis (Spitzer-Rimon et al., 2010). Like *ODO1, EOBII* encodes an R2R3-type MYB transcription factor. Suppression of *EOBII* significantly decreased the emission of most floral volatiles. Up-regulation and down-regulation of *EOBII* transcription increases and decreases the transcripts of many key enzymes in the FVBP pathway. As with *ODO1, EOBII* does not affect floral anthocyanin production. EOBII also directly up-regulated the expression of *EOBI*, a transcription factor gene closely related to *EOBII* (Spitzer-Rimon et al., 2012). *EOBI* suppression also reduces floral scent emission. Mechanistically, EOBI directly binds and activates the *ODO1, PAL*, and *IGS* promoters to regulate scent production (**Figure 1**).

A transcriptional enhancer region in the *ODO1* promoter was identified (Van Moerkercke et al., 2011). Mutations in putative MYB binding sites of this region decreased the overall promoter activity by 50%. *EOBII* bound to the MYB binding sites and enhanced *ODO1* promoter activity (Van Moerkercke et al., 2011). Polymorphisms that exist in the MYB binding sites in the *ODO1* promoter between the highly fragrant cultivar Mitchell and the non-fragrant cultivar R27 account for differential *ODO1* expression levels and scent emission levels in these cultivars. In addition, a hint of a potential negative element in the pathway was indicated, as a conserved *cis*-regulatory element known as Evening Element (EE) was present in the *ODO1* promoter. EE is a binding site for the clock transcriptional repressors *LATE ELONGATED HYPOCOTYL* (*LHY)* and *CIRCADIAN CLOCK ASSOCIATED 1* (*CCA1*) in *Arabidopsis thaliana* (Harmer, 2009).

These three transcription factors function as activators that broadly regulate the enzyme gene expression in the FVBP pathway. There is also a specific repressor, *PhMYB4* (Colquhoun et al., 2011). Repression of *PhMYB4* increased expression of both *CINNAMATE 4-HYDOXYLASE* (*C4H1* and *C4H2*) genes, which encode enzymes that modify *t*-cinnamic acid into *p*-coumaric acid. The increase in *C4H* expression in *PhMYB4* silenced lines likely accounts for the corresponding increase in the emission of eugenol and isoeugenol, which are derived from *p*-coumaric acid. Thus, *PhMYB4* is a negative regulator of eugenol and isoeugenol emission. A diurnal rhythm, similar to that of *ODO1*, was observed in *PhMYB4* expression.

## Clock Mechanisms Regulating Floral Scent Metabolism

Directly following the identification of putative binding sites of the clock components in the promoter of *ODO1* (Van Moerkercke et al., 2011), the first petunia clock gene, *Petunia hybrida LHY* (*PhLHY*), was identified (Fenske et al., 2015). Similar to its homologs in other plants, *PhLHY* peaks around dusk (**Figure 1**). Constitutive expression of *PhLHY* leads to an almost complete loss of scent emission, as well as a corresponding decrease in the expression of many FVBP genes (*ODO1, EPSPS, CM1, ADT, PAL*, etc.). Reducing the expression of *PhLHY* advanced the phase of scent emission from night to afternoon as well as FVBP gene expression. In addition, the direct binding of PhLHY to the EEs in the *ODO1* promoter was demonstrated. PhLHY also bound to the EE sequences of *EPSPS* and *IGS* promoters. Based on the results of the expression analysis, it seems possible that PhLHY can bind to other genes in the FVBP pathway and control the expression phase of these genes. As our current understanding of the transcriptional regulation of the FVBP pathway is still incomplete (**Figure 1**), a further investigation into the relationship of PhLHY with the FVBP transcription factors is awaited.

At the same time, similar results were observed in tobacco (*Nicotiana attenuata*; Yon et al., 2015). Repression of the *N. attenuata* homolog for *LHY, NaLHY*, caused a shift in scent release to an earlier phase, similar to the case in petunia, and repression of *N. attenuata ZEITLUPE* (*NaZTL*) led to a dramatic decrease in floral emissions. In addition, the same study explored clock effects on other important aspects of pollinator attraction: floral opening and the angle of flower position against the horizon. In long-day conditions, *NaLHY*-silenced lines showed a 2 h advance in floral opening time and flower angle changes, while *NaZTL*-silenced lines showed no clear change in timing, but a significant reduction in overall flower scent emission, limited opening and a change in position. In continuous light, *NaLHY*-silenced flowers started opening 4 h earlier than wild-type flowers, and the circadian period length of opening was approximately 1 h shorter than that of wild-type flowers, indicating that the role of LHY in the tobacco circadian clock is similar to that in the *Arabidopsis* clock.

While the latest findings on *LHY* and *ZTL* provide additional insight into scent regulation, further inquiry into the relationship that *LHY*/*ZTL* and other clock genes share with scent metabolism is called for, once more complete genomic information is available for these plants.

## Conclusion

Temporal expression of scent appears to be primarily regulated through manipulation of the timing of transcriptional regulators in the metabolic pathway. Regulating the expression of *ODO1* has a significant effect on floral scent emission, establishing its position as a master regulator of FVBP synthesis. Most recently, two independent research groups showed that the clock gene *LHY* set the pace of floral scent emission in two *Solanaceae* species (Fenske et al., 2015; Yon et al., 2015). LHY directly represses *ODO1* and likely other FVBP genes in the morning, restricting the expression of FVBP genes to the evening in petunia. With these findings, we are beginning to understand how the molecular clock regulates the timing of scent emission.

Many questions remain unanswered. For example, what is the role of other possible clock gene homologs on the regulation of scent emission? Are the mechanisms found in these two *Solanaceae* species conserved in other plants? Could changes in clock gene function/activity contribute to changes in pollinator choice during evolution? Finding the answers to these questions would bring us a more comprehensive and broader view of the regulatory networks for daily scent emission.

Most of the research into the regulation of scent emission has focused on transcription and volatile analyses. Thus, any circadian analysis of enzyme abundance and/or activity, together with metabolomic profiling, within the FVBP pathway would also provide further understanding of floral scent regulation. Understanding the mechanisms of floral scent release will hopefully allow for manipulation of the timing of floral scent release. In addition to applications in the floriculture industry, knowledge obtained from this type of work may allow us to generate “designer crops” which could facilitate the utilization of new pollinator populations to increase the success of reproduction under wider ranges of environmental conditions.

## Author Contributions

All authors listed, have made substantial, direct and intellectual contribution to the work, and approved it for publication.

## Conflict of Interest Statement

The authors declare that the research was conducted in the absence of any commercial or financial relationships that could be construed as a potential conflict of interest.
